# EEG connectivity features associated with fibromyalgia revealed by machine learning

**DOI:** 10.3389/fpain.2025.1704444

**Published:** 2026-01-15

**Authors:** Jean Li, Jeremiah D. Deng, Divya Adhia, Matthew Hall, Ramakrishnan Mani, Dirk De Ridder

**Affiliations:** 1 Department of Surgery and Critical Care, University of Otago, Dunedin, New Zealand; 2 School of Computing, University of Otago, Dunedin, New Zealand; 3 School of Physiotherapy, University of Otago, Dunedin, New Zealand

**Keywords:** AI, EEG, fibromyalgia, machine learning, pain

## Abstract

**Introduction:**

We present connectivity-based features associated with fibromyalgia, derived from raw EEG data at the sensor level.

**Methods:**

These connectivity features were identified through a data-driven method, employing machine learning. We carried out some automatic, moderate pre-processing and extracted spectral connectivity features. Machine learning experiments then followed, employing feature importance analyses and feature selection techniques for building high-performing classification models; finally, based on robust cross-validation and test evaluation, we obtained the features associated with fibromyalgia. The raw EEG signals from 463 participants are used in the primary analysis. An external dataset that consists of 48 participants is used to validate the identified connectivity features.

**Results:**

Five features in the gamma band (Fz-Cz, Pz-P4, Fz-C3, Cz-P4, and Cz-Pz) were able to objectively detect the presence or absence of fibromyalgia with an accuracy of 99.57%. The identified connectivity features associated with fibromyalgia also show promising results on EEGs that are collected using a different type of device.

**Discussion:**

EEG-based functional connectivity features associated with fibromyalgia, identified using machine learning in the gamma band at the sensor level, can distinguish between fibromyalgia participants and healthy controls with 99.57% accuracy. These findings advance our understanding of the brain-based mechanisms of fibromyalgia and provide novel targets for future non-invasive neuromodulation and neurofeedback trials. However, future studies need to replicate these findings in independent EEG datasets in people with fibromyalgia as well as compare with other clinical populations.

## 1 Introduction

Fibromyalgia, formerly known as neurasthenia and fibrositis [1], is a chronic primary pain condition that is characterized by widespread musculoskeletal pain accompanied by secondary symptoms, including fatigue, and disturbances in cognition, sleep, mood, and dysautonomia [2, 3]. Indeed, 84% of fibromyalgia patients suffered one or more traumatic events prior to the onset of pain [4]. The diversity of emotional, cognitive, and autonomic symptoms present in fibromyalgia has resulted in heated debates over whether fibromyalgia even exists. This is exemplified by comments on published papers such as “fibromyalgia and the medicalization of misery” [5], “fibromyalgia- real or imagined?” [6, 7], or “pain is real; fibromyalgia isn’t” [8].

Currently, fibromyalgia is diagnosed based on the 2016 ACR/EULAR criteria, which use patient-reported measures of widespread pain and symptom severity, along with confirmation of generalized pain and symptoms persisting for at least three months. Diagnosis is made through a combination of these validated self-reported symptom scores and clinical evaluation. An objective biomarker that could help diagnose fibromyalgia is currently lacking, and EEG-based biomarker studies in fibromyalgia remain scarce. Cortical-based alterations observed in people with fibromyalgia could be a diagnostic biomarker. Brain alterations have been demonstrated in people with fibromyalgia, in particular, heightened activity in the salience network (amygdala, cingulate cortices, insula) and somatosensory cortex [9]. These changes may be associated with chronic pain or other symptoms of fibromyalgia. Such changes could predict the presence of fibromyalgia with 93% accuracy, suggesting it is a typical brain signature associated with fibromyalgia that can be detected by artificial intelligence [10], clearly demonstrating it is a brain-related disorder and thus exists. Neuroimaging in fibromyalgia suggests decreased descending inhibition, with decreased activity of the pregenual anterior cingulate cortex (pgACC) [11–15]. Contemporary evidence-based guidelines for fibromyalgia, including those from EULAR and NICE, recommend non-pharmacological interventions—such as patient education, graded exercise, and psychological therapies—as the foundation of management. Pharmacological treatments (e.g., selected antidepressants or anticonvulsants) may be considered for some individuals, but opioids are not recommended for fibromyalgia or chronic primary pain. The high burden of disability in this population suggests that current treatments are inadequate [16, 17]. A key challenge in diagnosing fibromyalgia is the absence of clinically validated biomarkers. Although proposed brain signatures derived from stimulus-based fMRI paradigms show promise [10], this expensive and labor-intensive approach can only be conducted in highly specialized neuroimaging centers, limiting feasibility and preventing its adoption in routine clinical practice. Furthermore, the question remains whether such a stimulus-driven fMRI approach is optimal, as it reflects brain activation [18]. But if fibromyalgia is an emergent property of multiple brain networks [19, 20], then looking at connectivity may be more optimal than looking at activity at focal brain areas.

A user-friendly, clinically applicable system needs to be able to pick up the neural fibromyalgia signature on raw, unprocessed data in an automated way on an affordable device. The need to utilize raw EEG arises from various factors. Firstly, manual cleaning is costly, laborious, and time-consuming. To develop a simple and cost-effective pain-detecting method, it is necessary to bypass this manual cleaning step. Secondly, while the exclusion of artifacts has the potential to aid in the interpretation and analysis of EEG recordings, it comes at the cost of inevitable data loss in the cleaning process. At times, the exclusion of contaminated EEG segments can result in the loss of crucial information [21]. Previous studies have suggested that good quality can be achieved with raw EEG data despite the higher noise level [22] and that removal of ocular and muscle artifacts can even hamper the performance of machine learning technologies [23].

Spectral-based functional connectivity features can capture functional connectivity, defined as the strength of temporal correlation between brain regions of interest (ROIs). Recent work has demonstrated the utility of functional connectivity in EEG pain research, suggesting that this approach may outperform attempts to isolate a single pain-generating network [24]. These findings highlight the potential value of connectivity-based EEG metrics for characterizing pain-related neural processes in fibromyalgia. Accordingly, this study aimed to identify raw—i.e., not manually preprocessed—EEG features that are associated with fibromyalgia using spectral-based functional connectivity between sensors, rather than activity derived from individual sensor sites.

## 2 Materials and methods

### 2.1 Study design

This was a cross-sectional observational study using previously collected EEG data to identify sensor-level functional connectivity-based EEG features associated with fibromyalgia. All analyses were conducted using data-driven machine learning methods, and the performance of the identified features was validated using an independent external dataset.

### 2.2 EEG datasets

The datasets used in this investigation were sourced from two different countries and settings: the neurosurgical department of the University of Antwerp (UZA), Belgium, and the University of Otago, New Zealand.

Fibromyalgia was diagnosed according to the ACR-90 (American College of Rheumatology, 1990) criteria for fibromyalgia [25]. The inclusion and exclusion criteria for the ACR 1990 Fibromyalgia diagnosis are as follows. Inclusion criteria: 1. widespread body pain (present in the left, right, upper body, and lower body, as well as axial bone pain), and 2. tenderness at 11/18 defined trigger points [25]. The pain should not be explainable by other diseases (exclusion criteria) such as inflammatory arthritis (rheumatoid arthritis, polyarthritis and systemic disorder, systemic lupus erythematosus), axial skeletal syndromes (low back pain syndromes and neck pain syndromes), osteoarthritis of knee or hand, nonarticular disorders (tendinitis and regional syndromes), arthralgia syndromes [25].

EEG datasets of patients with primary fibromyalgia syndrome (*n* = 184) and healthy participants (*n* = 279) were used for identifying the connectivity features associated with fibromyalgia. These data were collected at the neurosurgical department of the University of Antwerp (UZA), Belgium. The EEG recordings of fibromyalgia patients (156 females, 28 males) (denoted by “Fibr”) were anonymized and deidentified, i.e., detached from the clinical information. These EEG recordings had been previously used in published reports [15, 26–33]. Similarly, the anonymized and deidentified EEG recordings of the healthy controls (173 females and 106 males; 17–89 years of age) (denoted by “HC”) have been used in previously published reports [34–37]. All participants have given informed consent for their EEG recordings to be used for EEG research purposes in accordance with the ethical standards of the Helsinki Declaration (1964), and the data were collected under the approval of IRB UZA OGA85. All the “Fibr” and “HC” data were acquired using the Mitsar 19-electrodes system in the standard 10–20 international placement (Fp1, Fp2, F7, F3, Fz, F4, F8, T3, C3, Cz, C4, T4, T5, P3, Pz, P4, T6, O1, O2). The EEG data from these two groups were acquired using physically different devices of the same model of Mitsar-201 amplifiers. A linked-ears reference was used, and electrode impedances were checked to remain below 5 kΩ. These EEG recordings were obtained in a fully lit room with each participant sitting upright in a small but comfortable chair. Recordings were obtained under eyes-closed resting-state conditions, sampled at 500 Hz, with no filter applied during acquisition. Artefact minimization was achieved by instructing participants to reduce eye blinks and muscle movements. This protocol ensured high-quality EEG, suitable for spectral and functional connectivity analyses.

An external dataset from the University of Otago, New Zealand, was used to validate the performance of the identified connectivity features associated with fibromyalgia on EEG recordings collected with a different type of device. This dataset comprises resting-state EEG recordings from 24 females (aged 22–75) diagnosed with fibromyalgia and 24 healthy control females (aged 18–52). Recordings were obtained under eyes-closed resting-state conditions. Ethical approval was obtained from the Health and Disability Ethics Committee, New Zealand (19/CEN/179 for the healthy control group, 2023EXP15164 for the fibromyalgia group). All participants provided written informed consent for their EEG recordings to be used for research purposes. In this external dataset, data from both groups were collected using a Compumedics Neuroscan device with the 64-electrodes placed in the standard 10-10 International placement. The demographic and clinical characteristics of the participants used as the external dataset are presented in Supplementary Tables S1, S2.

### 2.3 Pre-processing

All EEG recordings went through an automatic pre-processing procedure to remove potential artefacts and reduce diversity between the datasets. Specifically, the first 10 s of the raw recordings were discarded to avoid the initial high noise. A notch filter at 50 Hz to attenuate power-line interference was applied to all data, followed by a high-pass filter of 0.5 Hz and a low-pass filter of 44 Hz.

From the “HC” and “Fibr” datasets, 47 participants (10%) were randomly selected to form the test set. Data from this test set were used solely to obtain the final test scores. All training and validation processes were conducted on EEG data from the remaining 416 participants, from which the features associated with fibromyalgia were derived.

Since the number of subjects in the healthy and fibromyalgia groups was imbalanced, even sampling directly from the collected data would have resulted in significant class imbalance, potentially hindering the effective training of machine learning algorithms. To address this issue, we applied data segmentation and sampling steps to create balanced datasets. Specifically, each subject’s recording was segmented into five 30 s sections. During cross-validation, three sections from each healthy participant and five sections from each fibromyalgia patient were used in the training set. To obtain the test score, 5 sections of each test subject were used regardless of the classes. Although each segmented EEG section was considered an independent sample for model training, all train/test splits were performed at the participant level prior to segmentation to prevent data leakage, meaning EEG from the same individual never appeared in both the training and validation sets, even if the signals were from different time segments.

### 2.4 Feature extraction

In this study, the EEG samples are represented by the spectral connectivity between channel pairs. For each training sample, the EEG stream is segmented into 1 s epochs. Within each epoch, it is assumed that the spectral measure is stationary. For every pair of channels, the coherence measures are computed across all epochs in each frequency. The MNE package [38] is applied to compute the spectral connectivity measures. In particular, for every pair of channels, the coherence across all epochs in frequency ω is computed according to Equation 1:

$$C(\omega )=E\left[\frac{\left|S_{\mathrm{xy}}(\omega )\right|}{\sqrt{S_{\mathrm{xx}}(\omega )S_{\mathrm{yy}}(\omega )}}\right],$$ (1)

where $S_{\mathrm{xy}}(.)$ is the cross-spectral density for channels x and y, $S_{\mathrm{xx}}$ and $S_{\mathrm{yy}}$ are auto-correlations, and E[.] denotes averaging over all epochs within a sample.

The mean value of the connectivity within delta (1–4 Hz), theta (4–8 Hz), alpha (8–12 Hz), beta (12–30 Hz), and gamma (30–40 Hz) was then adopted to represent each band. Each data sample is therefore represented by a feature vector of length 855 (171 channel pairs × 5 bands).

### 2.5 Feature selection and model training

Feature selection was carried out using a modified sequential floating forward selection (mSFFS) method [39], as it was proven to outperform other benchmark methods on EEG data [39]. We used a support vector machine (SVM) classifier as the wrapper for the cross-validation. 10-fold cross-validation was repeated 10 times to obtain the cross-validation score of each feature combination. In each of the 100 trials, the data points of 90% of the training set individuals were used to fit an SVM classifier, and then we used the rest 10% to obtain a validation score. The final cross-validation accuracy is the average validation score of all trials. Then we trained the final SVM models on the whole training set, using the best parameters found through cross-validation to obtain the training accuracy. The final SVM used a radial basis function kernel, with gamma selected through a grid search over 0.0001 to 30 and optimized to 5.1053.

The number of selected features to be used in modelling will contribute to the complexity of the model, which may affect its generalization ability. We used cross-validation in deciding the optimal number of features because cross-validation is an established, reliable approach in model selection [40]. After assessing the performance using 1 to 20 features individually, the feature set chosen corresponds to the point on the cross-validation accuracy curve where an increase in the number of features ceases to yield significant improvement.

### 2.6 Feature importance analysis and data visualization

SHAP (SHapley Additive exPlanations) [41] was used to assess the importance of features, as SHAP is a widely used, powerful tool to assess the importance of features in learning models and improve their interpretability. Ranking the features by the mean absolute SHAP values informed us in choosing the ROI and the gamma band.

To visualize the data distribution, we employed the t-SNE algorithm [42] to embed the high-dimensional feature data into a 2-dimensional coordinate system. We used the Scikit-learn [43] library for the implementation of t-SNE in this study.

### 2.7 Cross-domain validation

Although the features associated with fibromyalgia were found on EEG recordings collected through a 19-channel device, we tested them on the external data that were collected using a 64-electrode device to check the robustness of these features. For consistency with the original 19-channel configuration, we selected from the 64-electrode dataset only the 19 electrodes that directly corresponded to the main 19-channel dataset. Recordings from the remaining electrodes were not included in subsequent analyses.

Most machine learning methods assume that training and test data are drawn from the same feature space and underlying distribution. When this assumption is violated, as in cross-domain evaluation, performance estimates may become unreliable or misleading [44–46]. Accordingly, a model trained on data collected using a 19-channel device cannot be assumed to generalize to data collected using a 64-channel device when the divergence between the two acquisition systems is substantial. Therefore, instead of directly testing the trained model on the external dataset, a new model was trained directly in the target domain using the identified features associated with fibromyalgia and abandon the source domain data. In addition to this direct training approach, we also adapted the model trained on the source domain data to the target domain data using transfer learning. This approach aimed to leverage knowledge learned from the source domain to improve performance in the target domain, under the assumption that shared patterns or features may exist across domains.

In each of the approaches, stratified cross-validations were carried out. Given that there are 24 participants in each group, the stratified cross-validations are divided into 2 to 24 folds, generating 23 mean cross-validation scores with an average training sample size ranging from 24 to 46 subjects. During each cross-validation split, data in one fold is held out for testing, and the other folds are used as the target domain training data.

In direct training, a new SVM classifier was trained on the target domain training samples in each fold, using the identified features associated with fibromyalgia as the feature representation.

In transfer learning, the labels from the training samples in the target domain were used to tune the original classification model trained on the source domain data. TrAdaBoost [47] is implemented to transfer the model trained using the identified features on the source domain data, to the training data from the target domain. Then this tuned model was tested on the hold-out fold in the target domain.

## 3 Results

### 3.1 Initial feature ranking

We included connectivity features from all sensor locations and frequency bands and employed the SHAP values to rank their importance in detecting fibromyalgia.

**Figure 1:**
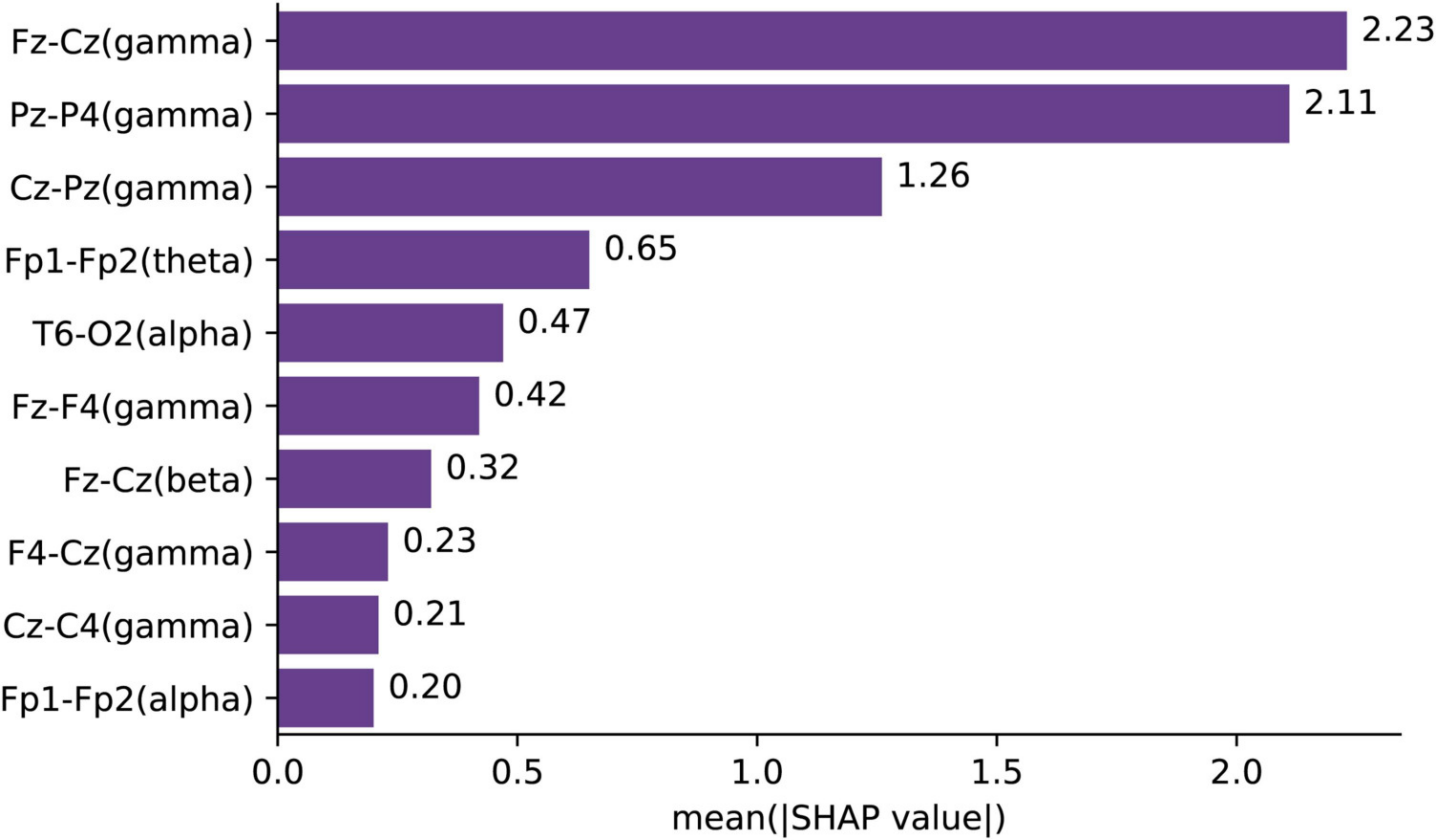
Mean absolute SHAP values of top-ranked features in all regions all bands.

155 of the 855 connectivity features report a mean absolute SHAP value above zero. The top 10 features in rank are Fz-Cz(gamma), Pz-P4(gamma), Cz-Pz(gamma), Fp1-Fp2(theta), T6-O2(alpha), Fz-F4(gamma), Fz-Cz(beta), F4-Cz(gamma), Cz-C4(gamma) and Fp1-Fp2(alpha). Figure 1 presents the mean absolute SHAP values of these features. It is shown that the features with the highest mean absolute SHAP values predominantly reside in the gamma band.

### 3.2 Top features in ROI gamma band

**Figure 2:**
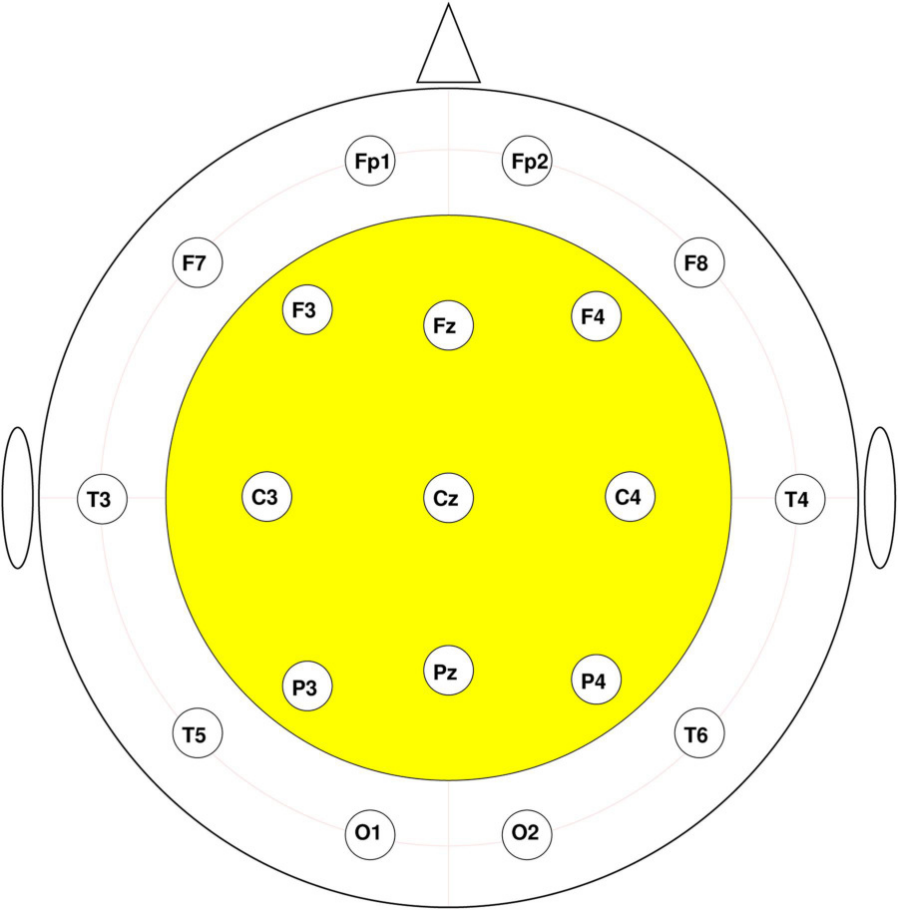
ROI highlighted in yellow.

For further analyses, we used the ROI comprising 9 central sensors: F3, Fz, F4, C3, Cz, C4, P3, Pz, and P4, as shown in Figure 2. This is because signals from the peripheral electrodes are likely to be severely interfered with by muscle activities due to possible facial tension [48]; and the initial feature ranking reveals that the most significant contribution to the classification model comes from the gamma connectivity features residing in this ROI.

With the ROI and gamma band selection put in place, we carried out SHAP analysis again on the restricted features. The top 10 features within ROI are Fz-Cz, Pz-P4, Cz-Pz, Fz-F4, Cz-C4, F4-Cz, Fz-C4, Cz-P4, P3-Pz, and Fz-C3.

### 3.3 The connectivity features associated with fibromyalgia

**Figure 3:**
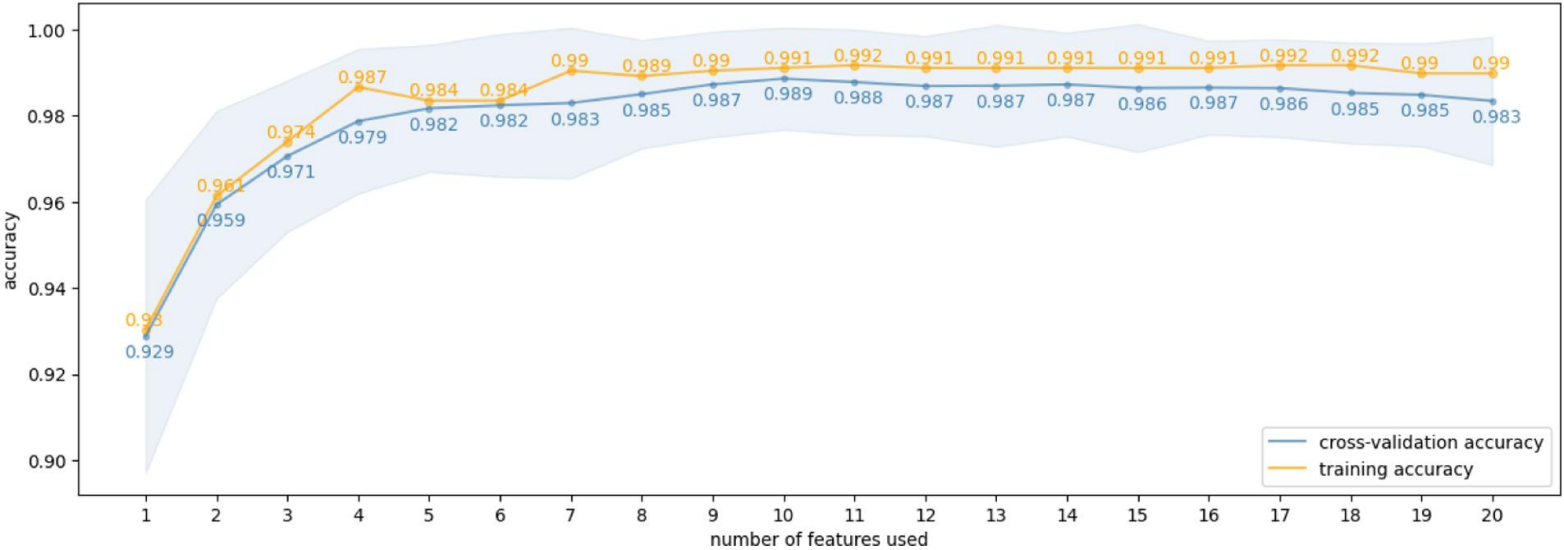
Classification accuracy scores when searching in ROI gamma band. Grey area gives the confidence intervals of the cross-validation scores.

**Figure 4:**
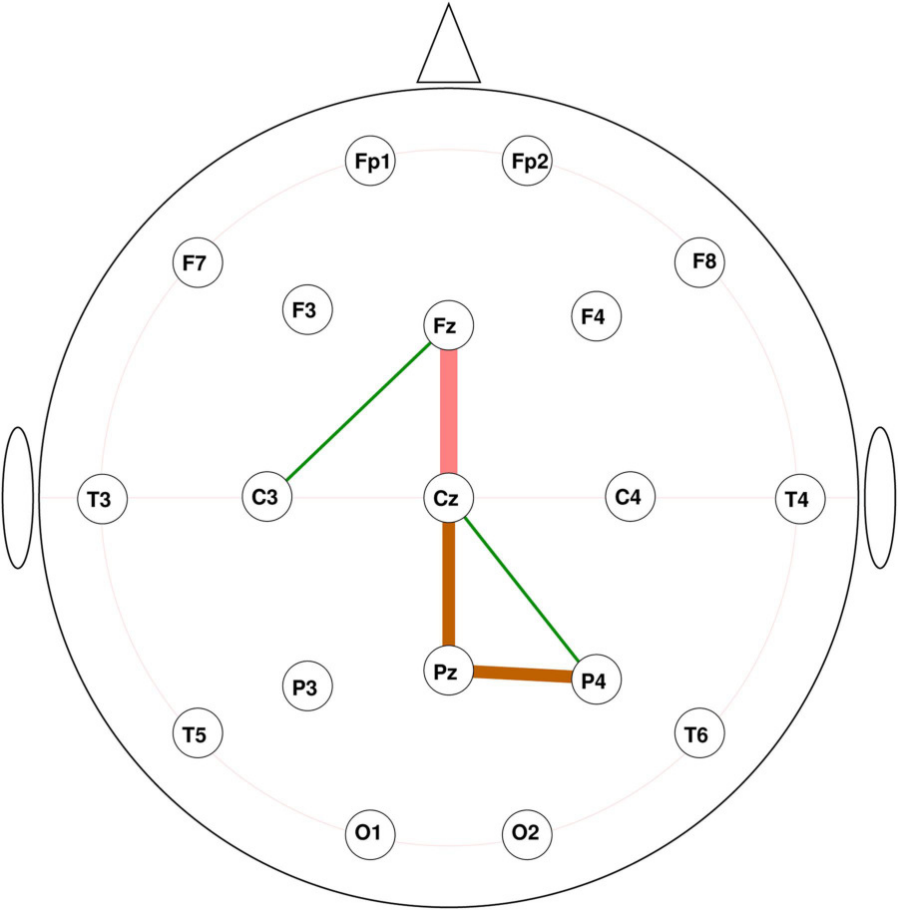
The connectivity features associated with fibromyalgia. The absolute mean SHAP values indicated in colour: green (<0.5), brown (≥1.0), pink (≥2.0); also by thickness.

**Figure 5:**
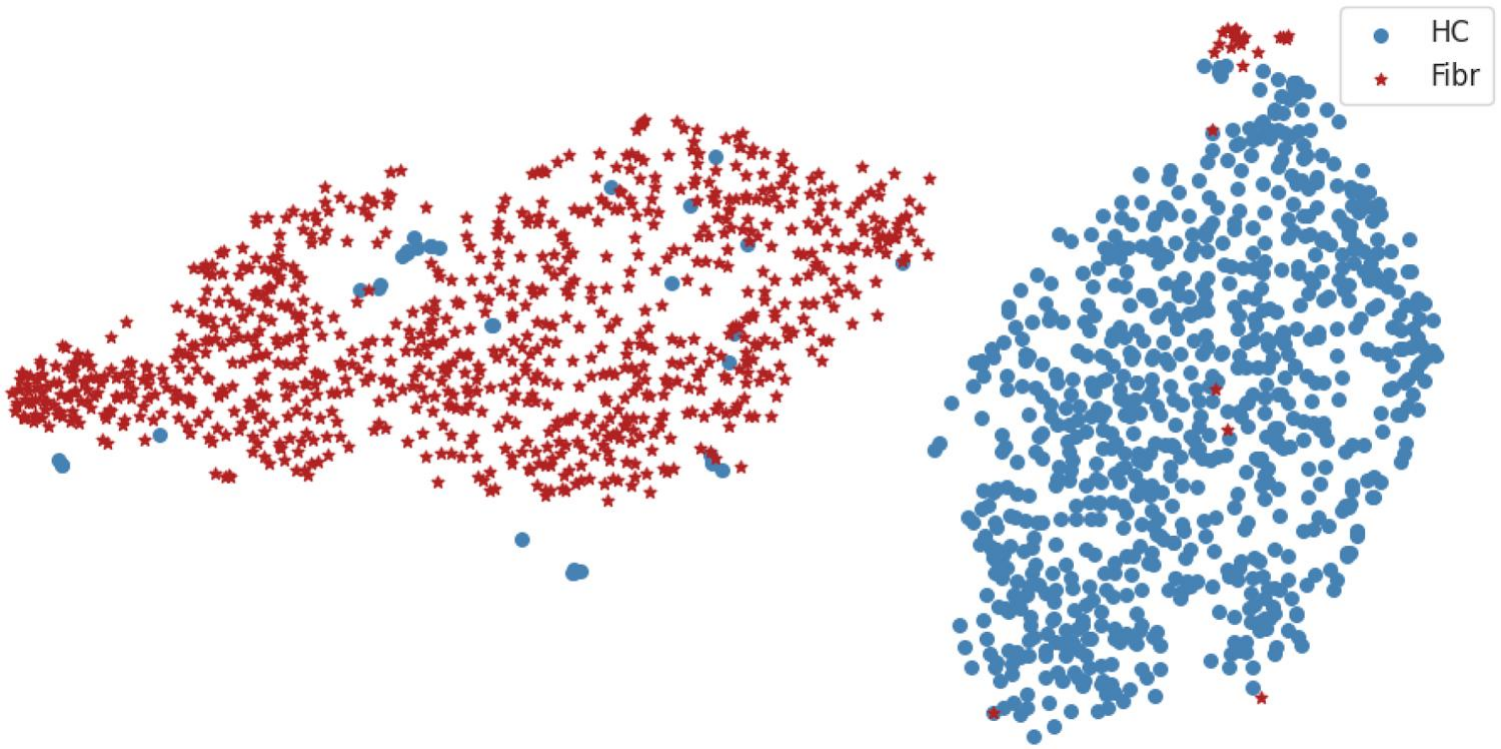
t-SNE visualization of all data using the connectivity features associated with fibromyalgia, with perplexity set to 100.

For the classification problem of fibromyalgia vs. healthy control, we produced feature selections of increasing sizes (1∼20) and obtained their corresponding cross-validation and training accuracies (Figure 3). The cross-validation curve indicated that a model with five features achieved a relatively high mean validation accuracy of 0.9821, with a standard deviation of 0.0148 and a 95% confidence interval of 0.9789–0.9846. These selected connectivity features are Fz-Cz, Pz-P4, Fz-C3, Cz-P4, and Cz-Pz (Figure 4). We use these 5 features as the final connectivity features associated with fibromyalgia, since using more features did not result in a significant improvement in cross-validation performance. When visualizing the distribution of data samples using the connectivity features associated with fibromyalgia, a clear separation between the fibromyalgia and healthy control groups is observed (Figure 5).

Using the selected features, the retrained classification model attained a test accuracy of 99.57% on the reserved test set, with precision and recall both at 0.9957, and an area under the ROC curve of 0.9964. The confusion matrix is presented in Supplementary Table S3.

To assess whether the classifier’s performance was significantly better than chance, we conducted a permutation test with 1,000 label shuffles. The mean accuracy across the permuted datasets was 0.4184 (SD = 0.0957). None of the permuted accuracies exceeded the observed accuracy, resulting in a permutation p-value of <0.001. These results indicate that the classifier’s performance is highly unlikely to be due to random chance, confirming the statistical significance of its predictive ability.

To evaluate the reliability of the predicted probabilities, we computed the Brier Score and Expected Calibration Error (ECE) for the classifier. The Brier Score, which measures the mean squared difference between predicted probabilities and true outcomes, was 0.0023, indicating highly accurate probability estimates. The ECE, representing the average absolute deviation between predicted probabilities and observed frequencies across bins, was 0.0142, demonstrating excellent average calibration of the model. Together, these metrics indicate that the classifier’s predicted probabilities are well-calibrated and can be interpreted as reliable estimates of event likelihood.

Sex distribution differed significantly between groups in the main dataset, with a higher proportion of females in the fibromyalgia cohort. To address potential sex-related confounding, a female-only sensitivity analysis was performed, given that fibromyalgia predominantly affects women. The cross-validation performance in the female-only subset (mean 0.9826, SD 0.0155, 95% CI: 0.9796–0.9857) was consistent with that of the full sample, indicating that the findings were not driven by sex imbalance.

**Figure 6:**
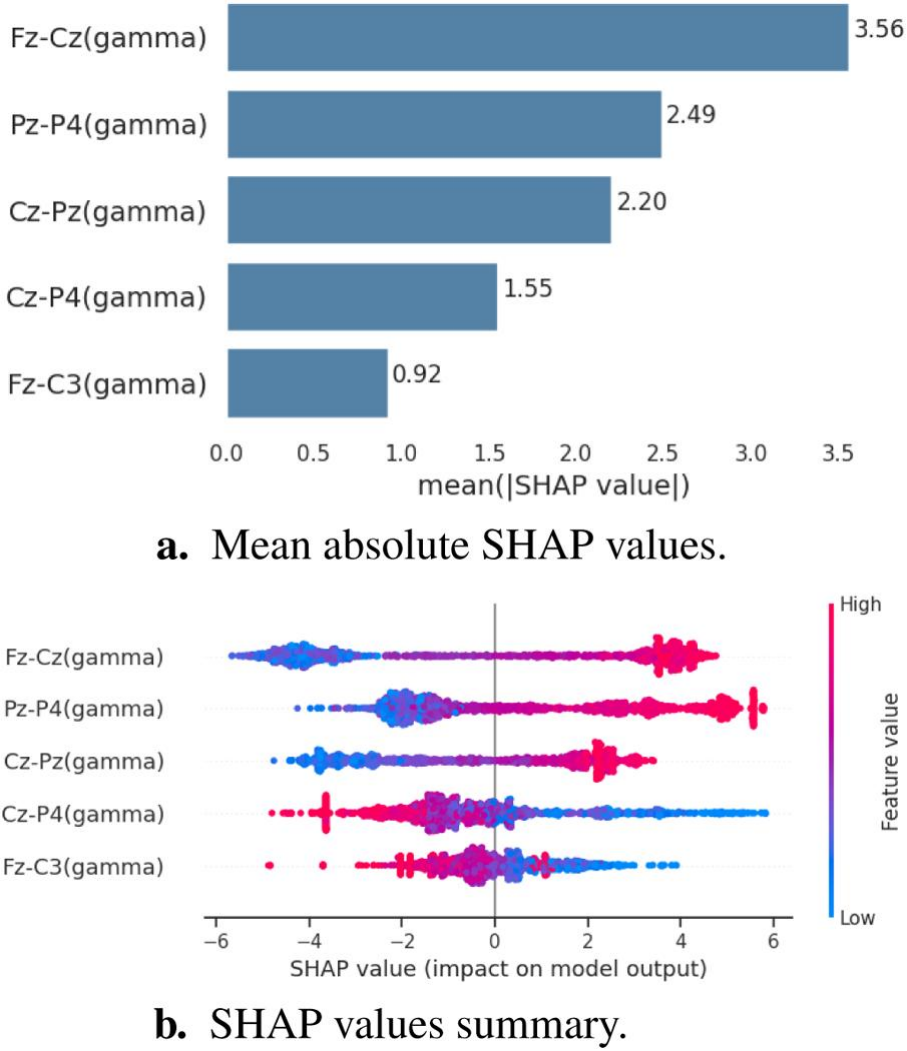
Graphical representations depicting the SHAP values for the connectivity features associated with fibromyalgia. **(A)** The mean absolute SHAP values across all samples. **(B)** The SHAP values distribution of all samples; higher positive values indicate a larger impact on fibromyalgia prediction, negative values indicate an impact on HC prediction; the colours are coded according to the connectivity strength.

To better understand the role of each connectivity feature, we performed SHAP importance analyses within the identified features associated with fibromyalgia. Figure 6A demonstrates the mean absolute SHAP value of each feature. According to their impact on prediction, the 5 connectivity features’ importance for the classification model are ordered as Fz-Cz, Pz-P4, Cz-Pz, Cz-P4, and Fz-C3. Figure 6B shows that the Fz-Cz connectivity is very important to the majority of the data sample, as most of them located on the far side from the origin; Pz-P4 connectivity contributes significantly to positive predictions, especially to a small group; Cz-P4 has thin, but longer tails, which indicates that this connectivity has an extremely pronounced impact on a small number of individuals, but is less important for majority.

### 3.4 Cross-domain validation results

**Figure 7:**
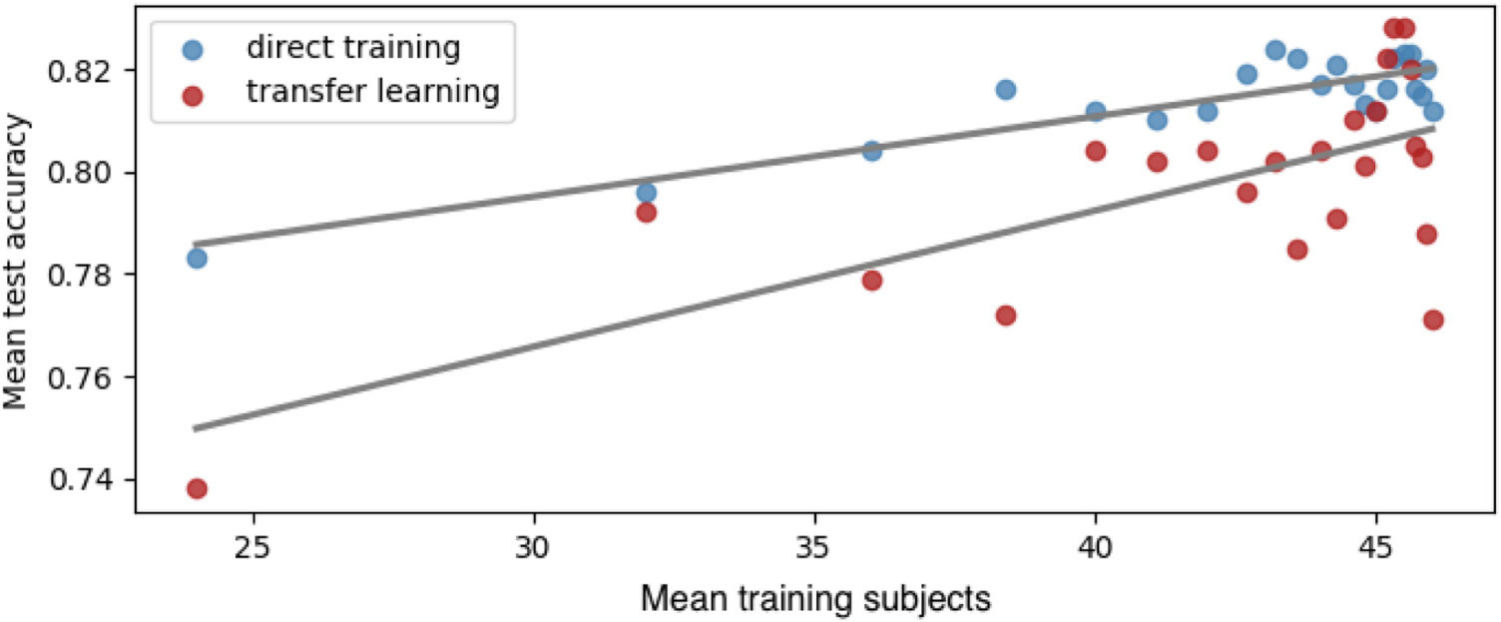
Cross-validation scores from transfer learning and direct training on fibromyalgia target set. The trend line is a linear regression model (order = 1) using the training sample size as the explanatory variable and the average test scores as the response variable.

Both transfer learning and direct training using the features associated with fibromyalgia achieved an accuracy of approximately 82%. Table 1 shows the average test scores of both transfer learning and direct training. These scores are fitted into a linear regression against the sizes of the training set with an order of one (Figure 7). Interestingly, the overall performance of direct training is better than that of transfer learning. However, this performance advantage is reduced with the increasing amount of training data.

**Table 1:** Test performance of the fibromyalgia target set.

Total cross-validation folds	Average training subjects	Transfer learning scores	Direct training scores
2	24.0	0.738	0.783
3	32.0	0.792	0.796
4	36.0	0.779	0.804
5	38.4	0.772	0.816
6	40.0	0.804	0.812
7	41.1	0.802	0.810
8	42.0	0.804	0.812
9	42.7	0.796	0.819
10	43.2	0.802	0.824
11	43.6	0.785	0.822
12	44.0	0.804	0.817
13	44.3	0.791	0.821
14	44.6	0.810	0.817
15	44.8	0.801	0.813
16	45.0	0.812	0.812
17	45.2	0.822	0.816
18	45.3	0.828	0.822
19	45.5	0.828	0.823
20	45.6	0.820	0.823
21	45.7	0.805	0.816
22	45.8	0.803	0.815
23	45.9	0.788	0.820
24	46.0	0.771	0.812

## 4 Discussion

Our data-driven analysis indicates that gamma band connectivity contributes strongly to discriminating fibromyalgia participants from controls. While gamma connectivity emerged as the most discriminative feature in this study, this does not exclude the involvement of other frequency bands, nor does it imply causality. High classification accuracy achieved using gamma band features should be interpreted with caution, as potential confounding variables and alternative explanations were not fully accounted for in this analysis. Previous resting-state EEG studies have documented additional alterations in fibromyalgia across alpha, beta, and theta bands, consistent with the thalamocortical dysrhythmia hypothesis, which posits disrupted thalamocortical circuits leading to abnormal oscillatory patterns and altered sensory processing [49–52]. Beta band abnormalities have also been associated with altered sensorimotor and cognitive processing in fibromyalgia [53]. These findings suggest that fibromyalgia is characterized by widespread dysregulation across multiple frequency bands, reflecting both tonic and phasic network dysfunction. Our results complement the existing literature by demonstrating that gamma band functional connectivity may capture network-level signatures relevant to pain processing, although we acknowledge that direct pain measures were not included in our models. Thus, gamma connectivity provides a potentially informative perspective on pain-related neural dynamics within the broader context of multispectral EEG abnormalities in fibromyalgia.

To contextualize why gamma band connectivity may be relevant in fibromyalgia, one conceptual framework is the Bayesian brain model, in which pain can be viewed as a signal arising from prediction errors within the brain’s internal generative model [19, 54]. Under the Bayesian brain theory, the brain is perceived as an internal generative machine utilized to predict sensory stimuli within the environment [55]. It actively samples from the sensory environment to seek validation for the sensory prediction [56, 57] and updates its model of the world to enhance future predictions. When the brain expects external stimuli from a changing environment, sensory predictions will be generated in the (low) beta frequency band, whereas prediction errors, for auditory [58–63], visual [64], painful and non-painful somatosensory stimuli [64, 65] will be reflected in the gamma band. Generally, beta activity reflects a status quo [66], in other words a correct prediction, whereas gamma activity reflects the unexpected, both positive and negative, prediction errors [67]. Therefore, if pain arises from a prediction error between the anticipated norm or baseline of the pain-free state and the sensed state, it is reasonable to anticipate that the transmission of the prediction error signal in the pain network occurs in the gamma band. Indeed, gamma oscillations have been shown to reflect pain perception in acute pain studies, i.e., related to acute pain induction [68–72] and pain intensity [72–74]. In addition, gamma oscillations have been used to characterize pain sensitivity [73], not only in humans but also in other species [72, 73]. Interestingly, this gamma band activity is measured only in the central electrodes of the EEG [73], analogous to what is found in this study. Furthermore, the pain-related central gamma activity is specific for pain, as it is not recorded in equally salient auditory, visual, and non-nociceptive somatosensory stimuli [73]. Importantly, a meta-analysis has demonstrated that this gamma band activity is present not only in acute phasic pain and tonic pain but also in chronic pain [72].

The identified features associated with fibromyalgia are based on connectivity rather than activity. This aligns with the concept that pain arises from an imbalance between pain-inputting and pain-inhibiting pathways within the neural network [19, 36, 54]. More specifically, the pain-inputting pathways consist of two main pain-provoking pathways: the medial and lateral pain pathways, which are distinct both anatomically and functionally [75–78]. The medial pain pathway consists of two different parts, the rostral to dorsal anterior cingulate (rdACC) and the anterior insular cortex. The rdACC of the medial pathway encodes the unpleasantness [76–80], in keeping with the definition of pain as unpleasant [81]. The anterior insula cortex of the medial pathway involves anterosuperior and anteroinferior sub-components encoding cognitive and emotional appraisals of pain experience [82]. The anterosuperior component correlates with the pain catastrophizing scale, i.e., the cognitive component of pain-related suffering [83]. The somatosensory cortex (SSC) is the main hub of the lateral pathway and processes the discriminatory/sensory components of the pain, such as pain intensity, pain localization, and pain character including burning, aching, etc. [75, 77, 84]. A descending pain inhibitory pathway, which is found to balance the two ascending pain pathways [85, 86], involves the rostral anterior cingulate cortex (rACC), the pregenual anterior cingulate cortex (pgACC), the periaqueductal gray, the parahippocampal area, the hypothalamus, and the rostral ventromedial brainstem [85–87]. Pain occurs when the pain input pathways outweigh the pain-inhibiting pathways. The concept of pain being an imbalance between networks suggests that the brain signature can be expected to be a network signature, i.e., a connectivity signature [19, 37, 54, 88, 89]. The sensors that are involved in the features associated with fibromyalgia may be related to connectivity coming from somatosensory cortices (Pz and P4) in the lateral pathway, and the anterior cingulate cortex (Fz and Cz) in the medial pathway. In other words, the identified features may be detecting the pain-inputting pathways. The regions of the sensor features that were found in this study overlap with some of the pain-predictive brain regions found in other studies [35, 90–92].

In the aspect of data space, EEG studies can be divided into two categories: the source space analysis and the sensor space analysis. EEG recordings are collected through sensor electrodes and are readily available for sensor space analysis. Source space analysis, however, necessitates the inversion of signals detected by sensors to retrieve the activity originating from specific brain regions. The analyses in this study are conducted on sensor space for several reasons. Firstly, from the perspective of entropy, there is no information gain from transferring signals from sensor space to source space. If the pathological patterns are present after the data are transformed into source space, they should also exist in the sensor space data. Secondly, the EEG inverse problem is an ill-posed problem due to its non-uniqueness and instability in finding a solution [93]. Although there are multiple methods (e.g., sLORETA, VARETA, S-MAP) that aim to solve the reverse problem, the reliability of these remains undetermined. This is because the extracranial nature of EEG means that each electrode can only collect a summation of information from multiple brain regions. As only the results of the summation can be observed, it is hard to determine if the final “10” comes from “2+8,” or “5+5.” This is why different reversing methods result in different solutions. Thirdly, while source-space analysis can, in principle, provide estimates of activity in specific brain regions, accurate source reconstruction typically requires high-density EEG (≥64 channels) to reduce uncertainty and improve spatial resolution. With only 19 channels, reliable source localization is not feasible, and results would be highly susceptible to the ill-posed inverse problem, leading to variable or spurious solutions. Performing analyses in sensor space allows for more robust and reproducible characterization of functional connectivity, while avoiding these methodological limitations. In addition to the reasons above, to pave the path for developing a fibromyalgia detection method that is clinically viable or creating better neuromodulation treatments, it is more practical and efficient to identify a neural signature at the sensor level. Lastly, conducting this study in sensor space becomes more logical after the initial analysis suggests that the gamma band should be the focal point. This is because analyzing in source space requires extra processing for solving the inverse problem to retrieve the brain sources, and this process requires that basal frontal, temporal, and occipital sensors are included in the recording. As these areas are prone to artifactual gamma band activity resulting from muscular activity [48], the analysis on a sensor level mitigates these potential false positive results.

The cross-domain validation suggest that the identified connectivity features associated with fibromyalgia not only perform well on a specific dataset but also has the potential to generalize effectively to data collected under different conditions. This indicates that the features associated with fibromyalgia are not device-dependent and exhibit overall potential. However, the external fibromyalgia data used for validation are collected using a device with a significantly larger number of channels compared to the original datasets on which the features associated with fibromyalgia were identified. The substantial differences in recording hardware, as well as the additional electromagnetic interference from these extra channels could potentially impact the performance of the identified features. Despite these potential performance-limiting factors, the performance remains acceptable, especially considering that the upward trend in performance suggests the current limitations may be due to the sample size. Therefore, it is reasonable to deduce that this study may only be presenting the lower bound of the potential test accuracy achievable on different datasets using the identified features associated with fibromyalgia.

There are several limitations to this study. First, the fibromyalgia predictions are based solely on correlational EEG data; no interventional validation was performed, and symptom changes following treatment were not assessed. Future prospective studies could adopt a longitudinal cohort design, collecting EEG data before diagnosis, during active fibromyalgia, and after symptom-modifying interventions. Given the need for large datasets for AI approaches, such studies will likely require multi-site collaboration. Secondly, no grading of the painfulness nor severity of the suffering is investigated. This should be added to future studies, ideally prospective in nature. Furthermore, due to data limitations, age and clinical characteristics were not included in the analyses. As such, the findings should be interpreted with appropriate caution. Future studies should aim to identify features associated with fibromyalgia in different age and sex groups. A possible taxonomy would be to group the female data into prepubertal, reproductive period, and postmenopausal groups, and investigate the features associated with fibromyalgia within each group. Lastly, the participant data were collected prior to the publication of the currently used 2016 ACR/EULAR fibromyalgia diagnostic criteria. As a result, diagnoses were based on the 1990 ACR criteria, which rely on tender point examination and may introduce gender bias. The 2016 criteria, which use patient-reported symptom measures, are now considered the standard. Future studies should apply the updated criteria, and our findings should be interpreted with this context in mind.

This study uses machine learning to identify features associated with fibromyalgia from raw resting-state EEG data at the sensor level. These features were characterized by connectivity patterns in the gamma band, particularly evident in central EEG electrodes (Fz-Cz, Pz-P4, Fz-C3, Cz-P4, and Cz-Pz). This EEG connectivity pattern represents an initial step toward identifying neural correlates of fibromyalgia that could, in the future, complement clinical assessments in a practical and non-invasive manner. While our results represent an early step toward understanding the neurophysiological correlates of the disorder, it is premature to consider this as a clinically validated biomarker. Future research is needed to determine whether such connectivity patterns could complement existing clinical assessments, potentially informing treatment monitoring or guiding neuromodulation strategies, while fully considering the multidimensional and subjective nature of fibromyalgia symptoms.

## Data Availability

The data analyzed in this study is subject to the following licenses/restrictions: The data that support the findings of this study are available upon request for research reasons. Requests to access these datasets should be directed to divya.adhia@otago.ac.nz.
