# Supplementary material for: EEG connectivity features associated with fibromyalgia revealed by machine learning
Source: Front Pain Res (Lausanne). 2026 Jan 15;6:1704444. doi: 10.3389/fpain.2025.1704444 (PMC12852373; doi:10.3389/fpain.2025.1704444)
Supplement: Supplementary file 1 [file Datasheet1.pdf]

Supplementary Material

1 SUPPLEMENTARY TABLES

Table S1. External dataset FMS patient characteristics. Abbreviations: FIQ = Fibromyalgia Impact Questionnaire; BPI Sev/Int = Brief Pain Inventory Severity/Interference; PVAQ = Pain Vigilance and Awareness Questionnaire; PCS = Pain Catastrophizing Scale; DASS S/A/D = Depression, Anxiety, Stress Scale; WHO-5 = Well-being Index; EQ5D HS/I = Health Status/Index.

Index	Age	Sex	FIQ	BPI Sev	BPI Int	PVAQ	PCS	DASS S	DASS A	DASS D	WHO-5	EQ5D HS	EQ5D I	Sleep
1	71	F	52.7	4.0	3.6	46.0	18.0	28.0	14.0	14.0	40.0	18.0	0.8	52.0
2	52	F	70.5	7.0	9.3	68.0	65.0	14.0	18.0	16.0	28.0	20.0	0.5	76.0
3	47	F	61.3	5.8	6.4	35.0	0.0	4.0	2.0	2.0	52.0	45.0	0.7	54.0
4	35	F	80.5	7.8	8.1	50.0	45.0	30.0	28.0	34.0	8.0	20.0	0.0	70.0
5	45	F	53.2	4.3	4.9	54.0	11.0	14.0	12.0	12.0	56.0	50.0	0.9	50.0
6	39	F	62.7	5.3	6.6	60.0	12.0	32.0	18.0	8.0	32.0	85.0	0.7	56.0
7	51	F	66.8	6.0	7.0	43.0	12.0	16.0	2.0	12.0	20.0	45.0	0.6	54.0
8	67	F	63.7	6.5	6.6	50.0	19.0	14.0	4.0	16.0	28.0	40.0	0.6	38.0
9	39	F	83.7	5.3	7.3	67.0	39.0	32.0	34.0	26.0	36.0	50.0	0.6	72.0
10	75	F	74.2	8.0	6.4	40.0	28.0	30.0	22.0	16.0	44.0	25.0	0.3	58.0
11	64	F	52.2	4.0	3.7	50.0	24.0	20.0	18.0	8.0	24.0	50.0	0.6	66.0
12	38	F	61.5	6.0	4.4	32.0	16.0	22.0	6.0	10.0	32.0	26.0	0.6	40.0
13	63	F	10.0	0.0	0.4	36.0	7.0	4.0	10.0	4.0	48.0	85.0	0.9	40.0
14	33	F	80.8	6.0	9.0	50.0	35.0	30.0	34.0	18.0	8.0	30.0	0.3	56.0
15	22	F	62.3	6.8	4.3	28.0	24.0	26.0	26.0	38.0	16.0	28.0	0.3	60.0
16	25	F	65.0	5.0	4.6	45.0	11.0	14.0	22.0	2.0	56.0	65.0	0.6	60.0
17	53	F	71.3	7.5	8.1	55.0	12.0	10.0	8.0	10.0	20.0	65.0	0.6	50.0
18	27	F	58.5	4.0	5.7	46.0	27.0	28.0	26.0	12.0	28.0	50.0	0.7	46.0
19	32	F	54.0	5.8	7.9	46.0	50.0	28.0	16.0	14.0	36.0	60.0	0.3	82.0
20	32	F	64.7	6.5	7.9	35.0	3.0	6.0	6.0	0.0	60.0	60.0	0.3	44.0
21	36	F	61.7	4.8	4.1	42.0	12.0	14.0	14.0	2.0	44.0	40.0	0.7	52.0
22	49	F	78.8	7.0	8.3	58.0	39.0	36.0	38.0	32.0	32.0	45.0	0.4	82.1
23	33	F	71.3	5.8	7.4	61.0	26.0	10.0	6.0	14.0	32.0	40.0	0.8	38.0
24	42	F/NB	48.0	3.5	7.3	49.0	20.0	26.0	15.0	24.0	24.0	60.0	0.5	40.0

Table S2. Externar dataset health control participant characteristics.

Index	Age	Sex	Other Conditions	Current Treatment	Medical Conditions
1	22	F	None	None	No
2	19	F	None	None	No
3	20	F	None	None	No
4	30	F	None	None	No
5	18	F	None	None	No
6	38	F	None	None	No
7	19	F	None	None	No
8	49	F	None	None	No
9	19	F	None	None	No
10	23	F	None	None	No
11	20	F	None	None	No
12	22	F	None	None	No
13	45	F	None	None	No
14	28	F	None	None	No
15	52	F	None	None	No
16	19	F	None	None	No
17	22	F	None	None	No
18	19	F	None	None	No
19	19	F	None	None	No
20	21	F	None	None	No
21	21	F	None	None	No
22	36	F	None	None	No
23	40	F	None	None	No
24	43	F	None	None	No

Table S3. Confusion Matrix

	**Predicted: Negative**	**Predicted: Positive**
**Actual: Negative**	139	1
**Actual: Positive**	0	95
